# Pupil size reflects successful encoding and recall of memory in humans

**DOI:** 10.1038/s41598-018-23197-6

**Published:** 2018-03-21

**Authors:** Michal T. Kucewicz, Jaromir Dolezal, Vaclav Kremen, Brent M. Berry, Laura R. Miller, Abigail L. Magee, Vratislav Fabian, Gregory A. Worrell

**Affiliations:** 1 Department of Neurology, Mayo Clinic, Rochester, MN USA; 2 Department of Physiology & Biomedical Engineering, Mayo Clinic, Rochester, MN USA; 3 Czech Institute of Informatics, Robotics and Cybernetics, Czech Technical University in Prague, Prague, Czech Republic; 4 Department of Physics, Faculty of Electrical Engineering, Czech Technical University in Prague, Prague, Czech Republic

**Keywords:** Cognitive control, Sensory processing, Human behaviour

## Abstract

Pupil responses are known to indicate brain processes involved in perception, attention and decision-making. They can provide an accessible biomarker of human memory performance and cognitive states in general. Here we investigated changes in the pupil size during encoding and recall of word lists. Consistent patterns in the pupil response were found across and within distinct phases of the free recall task. The pupil was most constricted in the initial fixation phase and was gradually more dilated through the subsequent encoding, distractor and recall phases of the task, as the word items were maintained in memory. Within the final recall phase, retrieving memory for individual words was associated with pupil dilation in absence of visual stimulation. Words that were successfully recalled showed significant differences in pupil response during their encoding compared to those that were forgotten – the pupil was more constricted before and more dilated after the onset of word presentation. Our results suggest pupil size as a potential biomarker for probing and modulation of memory processing.

## Introduction

Pupil size has been associated with cognitive processes underlying perception, attention and action for external stimuli. Pupil dilation was shown to indicate interest in the content of the presented visual stimuli, which revealed sex-specific differences^1^. It is also known to indicate general mental activity and correlate with task difficulty^2,3^. More recent studies have shown that high-resolution tracking of pupil size can be used to predict perception of specific stimuli^4^ and even the voluntary decisions about attending the stimuli^5^. In these experiments, pupil size alone was able to predict an overt decision about timing an action and a covert decision about choice of the stimulus^5^, suggesting a link between pupil responses and the higher-order brain systems supporting cognition, decision-making and/or execution of actions.

The anatomy and physiology of the brain pathways controlling pupil size have been well described^6^, and are known to involve both the autonomic and somatic nervous systems. Adrenergic and cholinergic neuromodulation has been implicated in the regulation of these pathways^5,7^ and, more generally, of the thalamo-cortical brain networks during states of sleep, wakefulness and cognition^8^. The tight link with these wide spread neuromodulatory systems inspired research into the relationship between the brain states, electrophysiological activities and the pupil response. Tracking pupil dilation was shown to correlate with transitions in the cortical state as measured in the intracellular membrane potential across multiple brain regions^9^. Furthermore, pupil size and these cortical arousal states were associated with slow and fast electrophysiological activities^9,10^ – low arousal and constricted pupil with low-frequency oscillations, compared with enhanced sensory responses, arousal and dilated pupil with high frequency oscillations. Hence, pupillometry has become an attractive tool for accessing information about the brain states and neurophysiological processes supporting sensory perception, attention and decision-making.

Less is known about pupil responses during memory processing. In a classic study using a short-term memory task pupil size was found to be proportionally increasing with the amount of information remembered and was correlated with task difficulty^3^. A recent report showed that, in addition to the amount of information held within memory, pupil size was correlated with accuracy of memory representations^11^. Several other studies have explored the pupil size as a predictor of recognition memory performance^12–15^. In these tasks a set of images is presented for a later recognition phase, in which the same set is presented mixed with new images for ‘old’ *versus* ‘new’ memory decisions. Pupil size was shown to be modulated not only by the emotional valence and novelty of the presented images, but also by the memory of the familiar ones (‘old/new effect’)^12,13,15^. Hence, pupillometry was proposed to provide a signal for ‘strength of memory’^13^, ‘memory retrieval’^15^, and ‘neural novelty’^14^.

It remains elusive, however, if pupil size can be used to predict successful encoding of freely recalled memory. In the recognition memory tasks, pupil responses are compared between either familiar or novel items that are presented for a memory-based decision. It would be important to know whether changes in the pupil size during memory encoding can predict subsequent free recall of an item without being presented for choice, and thus provide a biomarker for estimating likelihood of successful memory encoding. Brain activities measured using electrophysiological and neuroimaging techniques can be used to differentiate stimuli that are likely to be remembered from the ones that will be forgotten^16,17^. These techniques typically require invasive or expensive recordings of brain activity, and sophisticated tools for data acquisition and analysis. For instance, a recent study applied machine learning approach to predict memory encoding from invasive human recordings during free recall tasks^18^. A memory signal that can be easily accessed from tracking pupil size and thus by-pass the need for brain recordings would have large impact on the neuroscience research of memory functions and on development of new brain-machine interface technologies to modulate these functions. The biomarker signal could thus be used for e.g. responsive brain stimulation triggered during identified states of low likelihood of memory encoding. Therefore, we investigated pupil responses across different phases of a free recall memory task in human subjects as they encoded and recalled verbal information.

## Results

**Figure 1:**
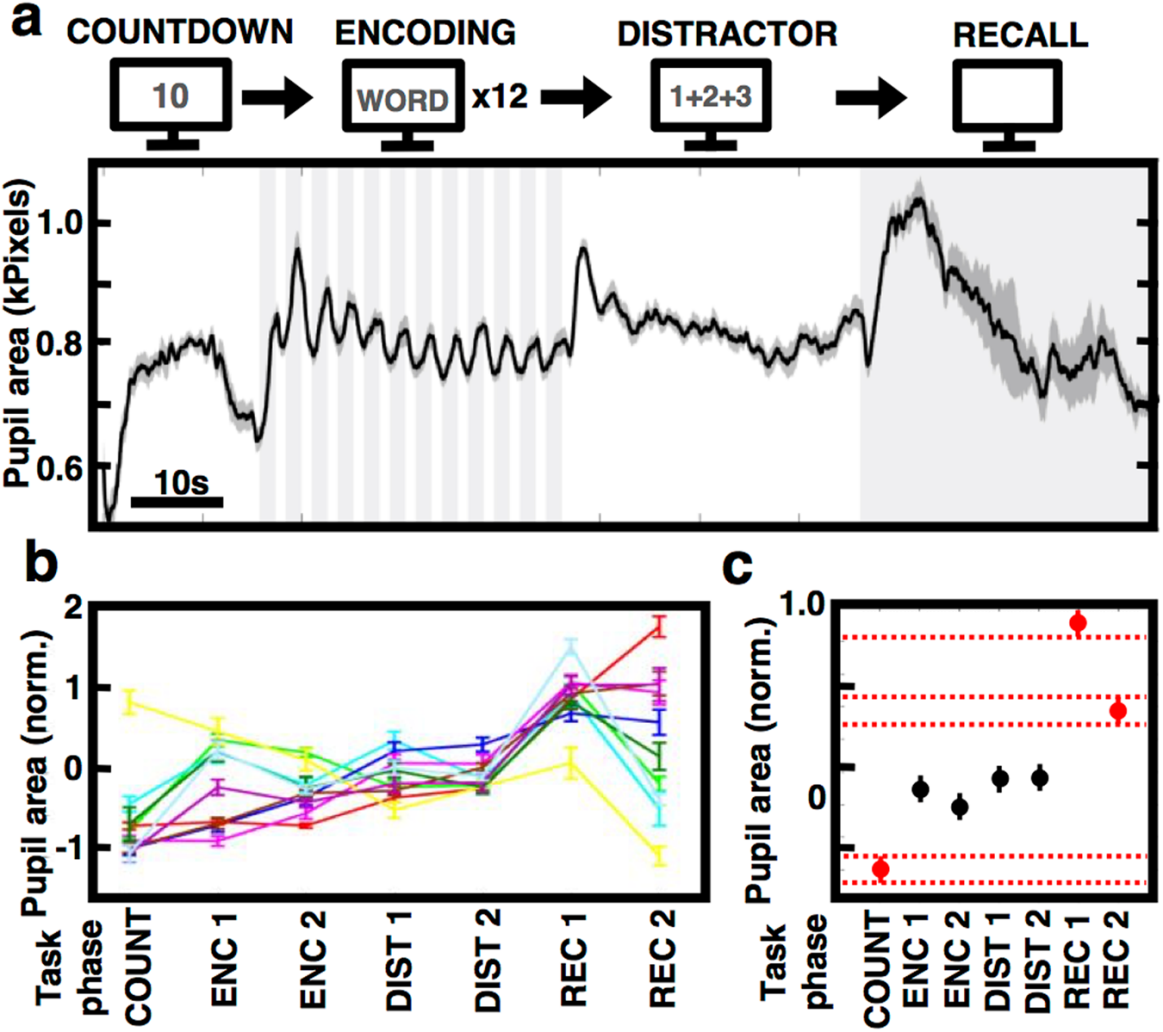
Pupil dilation is modulated by different phases of the free recall verbal memory task. (**a**) Trial-averaged changes in pupil size of one subject across four phases of the free recall task. Shaded areas mark epochs of word presentation on the screen and their recall with blank screen. Notice the consistent and stereotypical pupil responses across the trials revealing gradually increasing size in successive task phases. (**b**) Mean changes in pupil size are summarized in 12 s time bins of the four task phases for every subject (colors are different subjects). (**c**) Post-hoc ANOVA group comparison of means from the task phase bins (as in ‘**b**’) shows that pupil area was decreased during countdown and increased during recall. Red dotted lines are 95% confidence intervals. Note: the two phases are characterized by no cognitive load in the former and maximum load in the latter.

We employed a classic behavioral paradigm for free recall of verbal information to probe human memory encoding and recall^19^ with high-resolution tracking of gaze position and pupil size^20^. The memory task comprised of four successive phases of the encoding-recall procedure (Fig. 1a): ‘countdown’ from 10 to 1 with no memory load, ‘encoding’ of the words displayed individually one after another, ‘distractor’ task completing simple arithmetic equations to prevent rehearsing the word list and minimize the primacy and recency effects^19^, and ‘recall’ when the remembered words were vocalized in any order (see Methods for further details).

Pupil size was remarkably consistent across the entire experimental session and revealed robust changes in the absolute estimate of the area (Fig. 1a). These estimates were normalized for every subject within each encoding-recall procedure of a given word list and averaged in 12 s bins centered in the middle of each phase (encoding, distractor and recall phase were divided into half ‘1’ and ‘2’), showing a trend of increasing pupil size with the successive phases of the task (Fig. 1b). Analysis of variance confirmed a strong effect of the phase (ANOVA, F = 195.4, 6 d.f., p < 0.0001), no effect of the subject (F = 0.47, 9 d.f., p = 0.90), and a significant interaction between the phase and subject (F = 22.52, 54 d.f., p < 0.0001). Pupil size was the largest in the final two recall phases and most constricted in the first countdown phase (Fig. 1c), compared to any other phase of the task (Tukey-Kramer post-hoc comparison, p < 0.05). Since there was no memory component in the countdown phase and memory for words was gradually added and maintained along successive phases of the task, this general pattern suggests a correlation between cognitive load in the task and pupil dilation as previously proposed^2,3^.

### Pupil size during free recall of memory

**Figure 2:**
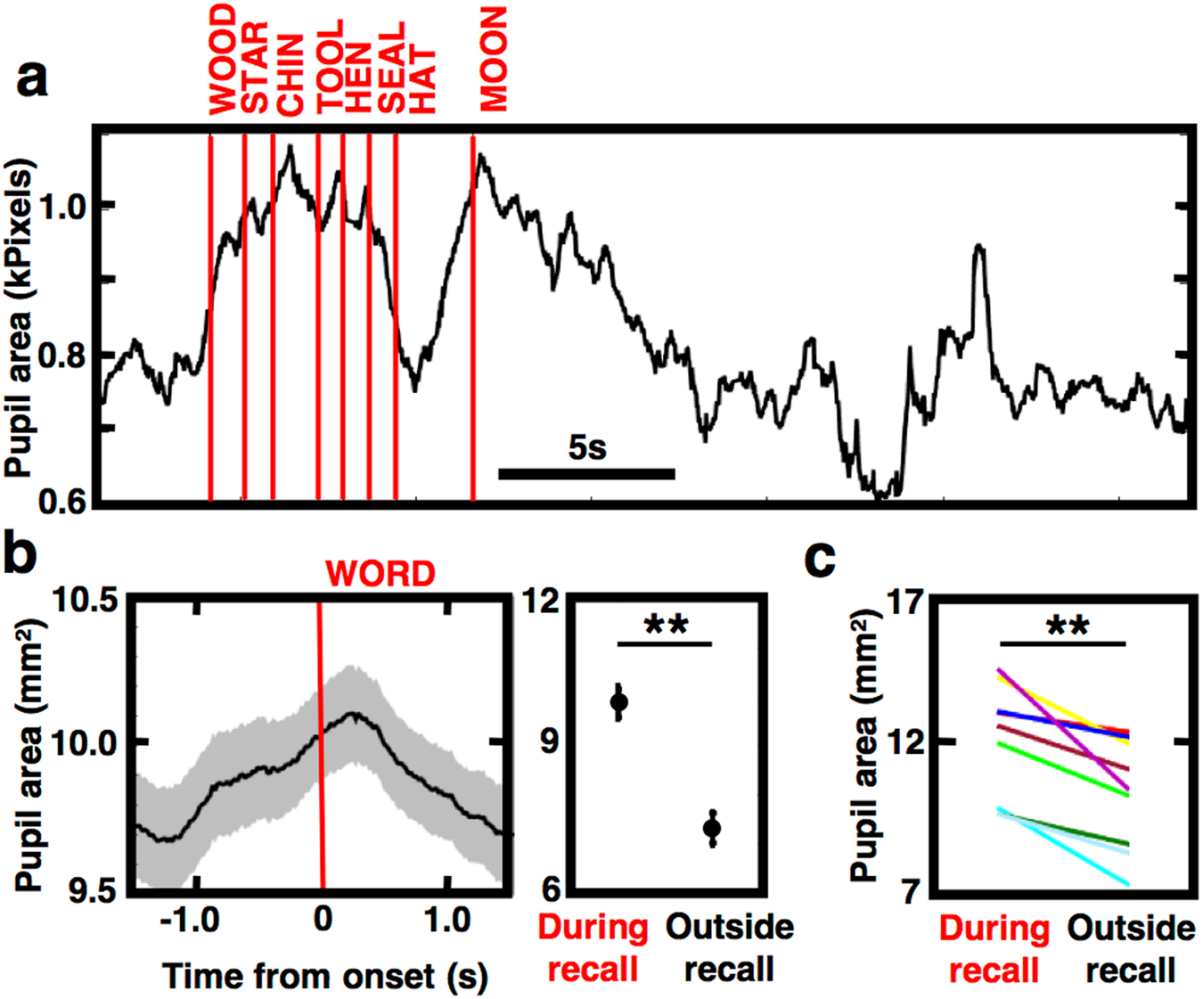
Pupil size is increased in response to free recall of remembered words. (**a**) Example of pupil area modulation during free recall of remembered words from one recall trial. Red lines mark the start time of word vocalization. Notice that recall of words is associated with pupil dilation with no changes in the screen display. (**b**) Mean pupil responses from all recalled word epochs in one patient are aligned to the onset of vocalization (left). Notice the consistent dilation starting before and peaking at the time of vocalization. Mean pupil area in ±1 s epochs around the word vocalization (‘during recall’) is significantly greater than in the remaining recall epochs (‘outside recall’) with no vocalization (**p < 0.01). (**c**) Across-subject comparison (colors are different subjects) of the pupil area in the two epoch types shows consistently more dilated pupil during recall of remembered words (*p < 0.05).

Assuming that pupil dilation correlates with cognitive load or effort in the task, it would be expected to be different at times when words are being recalled from memory and when they are not. We observed large pupil dilation at times when subjects were actively recalling words (Fig. 2a), which could not be attributed to any changes in the screen display (screen was blank during the entire recall phase) or lighting in the room. This increase in the pupil size started rising before the onset of the vocal response and gradually decreased afterwards (Fig. 2b), which does not exclude a possibility that the two may be related through a preparatory process initiated before the response. Recall epochs around this response (1 s before and after word vocalization) were characterized by greater pupil size as compared to the recall epochs outside of these vocalizations (Fig. 2b). This effect was significant in individual subjects (Fig. 2b) and on the group level (paired t-test, N = 9, p = 0.0024; one subject was excluded from this analysis based on proportion of time during recall with eye-tracking outside of the screen area – see Methods) with each individual subject showing a greater mean of the absolute pupil size in the word recall condition (Fig. 2c). Therefore, pupil size reflected a cognitive process associated with active recall of the encoded memory on the level of individual subjects and the whole group.

### Pupil response to encoding of remembered and forgotten words

**Figure 3:**
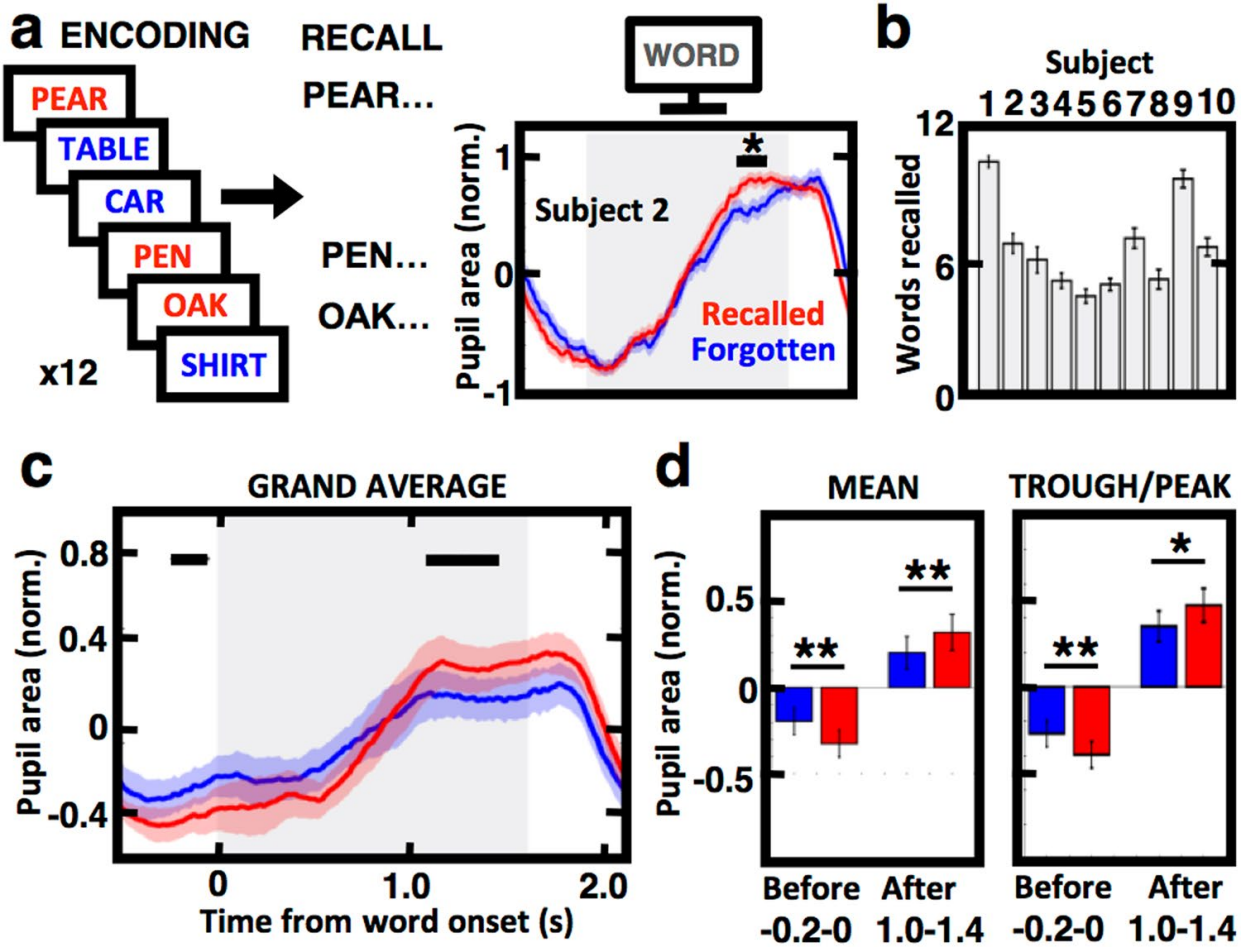
Remembered and forgotten words show different pupil responses during memory encoding. (**a**) Diagram on the left shows an example list of words presented in a sequence during encoding trials with subsequently recalled (red) and forgotten (blue) words. Mean pupil responses to presentation of words on the two trial types (right) in one example subject reveal more dilated peak response during encoding of the recalled words (horizontal bar below the asterisks indicates 50 ms bins with significant difference with p < 0.01). Shaded area marks the time of word presentation on the screen. (**b**) Mean memory performance of the ten subjects. (**c**) Subject-averaged pupil response to word encoding is presented as in ‘a’. Notice that pupil was more constricted on the recalled word trials just before the screen presentation, and more dilated at the peak response during encoding (bars indicate the time bins of the greatest difference). (**d**) Comparison of the subject means (left) and peak/trough values (right) in the epochs ‘Before’ and ‘After’ presentation onset (see ‘**c**’) confirms differential modulation of the pupil size between the trials with recalled and forgotten words (**p < 0.01, *p < 0.05 with Bonferroni correction for multiple comparisons).

To further investigate possible cognitive processes reflected by the pupil response, we compared the encoding period of words that were subsequently remembered and recalled to those that were not. Pupil responses were normalized for each word list by transforming the raw signal during the encoding phase into z-scores (see Methods). The normalized responses were then compared between the recalled and forgotten word conditions (Fig. 3a). Subjects showed a consistent pattern of response to word encoding – initial pupil constriction was followed by dilation peaking toward the end of word presentation on the screen, at which point the greatest difference between the two conditions was observed (Fig. 3a). Despite considerable variability in pupil response patterns and subject memory performance ranging from five to ten words recalled on average (Fig. 3b), the pupil response pattern revealed consistent trends across different subjects. The greatest difference between the two conditions was in the first 200 ms ‘before’ and 1000 ms ‘after’ the word onset with more constricted and dilated pupil in the recalled word condition, respectively (Fig. 3c). This subsequent memory effect was quantified by comparing mean values in these epochs as well as the peak and through for all subjects (Fig. 3d). Pupil size during encoding of subsequently recalled words had significantly lower mean (paired t-test, N = 10, p = 0.0044) and through (p = 0.0015) values before word onset, and significantly higher mean (p = 0.0021) and peak (p = 0.0121) values after the onset. Our findings suggest that pupil reaction right before and during presentation of the stimuli can be used to predict their subsequent memory recall.

## Discussion

Our results show that the signal sampled from tracking changes in pupil area contains information about the brain states and cognitive processes underlying memory encoding, maintenance and recall. In congruence with the previous reports^2,3,11^, we observed a general pupil size increase with mental effort and difficulty across the successive phases of the task. Task difficulty was increased from the encoding through the distractor phase of the task as the memory for words had to be maintained and freely recalled during the final phase when the pupil size was at its largest. There was a significant drop in the pupil size going from the first to the second half of the recall phase (Fig. 1), which can be explained by gradual ‘unloading’ of the actively maintained items from a memory buffer. Most of the words were recalled in the first half of this phase. Recalling a word was associated with ramping up of the pupil size that started before the time of vocalization (Fig. 2), which may be related to preparatory perceptual, cognitive or motor processing. In the encoding phase, pupil size was also consistently ramped up after presentation onset peaking at longer latencies above 800 ms (Fig. 3) when one would expect subject engagement in creating mental representations (e.g. visual depiction or words), active rehearsal, or other strategies employed for enhanced memorization. Both the gradual ‘macro-scale’ increase across the task phases and the ‘micro-scale’ pupil dilation around the recall and encoding of individual words suggest pupil size as an indicator of the processes engaged in storing, maintaining and retrieving information.

For an indicator of brain processes involved in these complex cognitive functions, pupil responses were found to be remarkably robust across subjects. Pupil responses varied between different subjects, showing patterns specific to a given individual. Despite these subject-specific differences, we still observed consistent changes in the pupil response both on the level of the task phases and presentations of individual words for encoding. The latter showed an initial constriction of the pupil size before the presentation followed by a later dilation during and beyond the interval of word display on the screen (Fig. 3). On the level of individual subjects, the mean and trough of the constriction, and the mean and peak of the dilation were different between words that were subsequently recalled and those that were not at very specific times of word encoding. Although such subsequent memory effect was reported in electrophysiological and brain imaging studies^16,17^, it would not be expected to be as consistent in its latency and across different subjects. The BOLD signal is limited in its temporal resolution, whereas the electrophysiological signals show variable latencies depending on the brain region and the frequency band analyzed^21,22^. In the same tasks, power changes in the gamma frequency bands revealed a similar pattern of decreased activity before word presentation and increased activity afterwards on the trails with subsequently recalled words^23^. Latency and magnitude of this electrophysiological subsequent memory effect was more variable than the pupil size responses and less generalizable. This study was also limited to a low number of subjects tested relative to the studies comparing brain activity. In spite of a low number of subjects and trials, and the individual differences in the tested group of subjects, there were still significant differences at the time of the trough and peak of the pupil response. Similar differences were observed with the gamma activity^23^, which altogether could reflect decreased encoding in preparation for word onset (pupil constriction and decreased gamma) followed by enhanced encoding during the presentation time (pupil dilation and enhanced gamma). High-resolution tracking of the pupil size, therefore, provides a new biomarker for memory processing, complementary to the currently used brain activity measures and advantageous in terms of its accessibility and robustness.

Pupil dilation and the electrophysiological measures of memory processing remain to be directly compared in a future study combining intracranial patient recordings with eye-tracking. Such recordings were so far only done in studies with non-human primates, which focused on tracking the gaze rather than the size of pupil^24–26^. Phase reset in low-frequency oscillations^24^ and increased incidence of high frequency oscillations, called the sharp-wave ripples^25,26^, were associated with memory performance and eye movements to remembered stimuli. Elucidating the relationship between the eye-tracking and electrophysiological measures will be critical to advance our understanding of these biomarkers and the brain mechanisms supporting memory processing. Eye-tracking can help to dissociate brain activities underlying memory processing from perception, attention and decision-making by following saccades, fixations and pupil dilation. Furthermore, specific brain activities can be correlated with specific eye-tracking features. For example, recent rodent studies correlated sharp-wave ripples in the hippocampus with pupil dilation and brain states of arousal and attention^9^. Similarly, sharp-wave ripples in primates were reported in response to the stimuli that were attended to with smaller saccades and longer fixations, which increased the probability of perceptual detection^26^. In another study, the sharp wave ripples occurring around the time of fixations on stimuli were shown to be indicative of their subsequent memory^25^. Human studies employing new techniques for recording these high frequency activities^27^ together with advanced high-resolution eye-tracking will shed more light on the underlying neuronal processes.

Our current study is limited in terms of what can be inferred about memory processes from the behavioral measure of pupil size responses in a free recall task. Other memory tasks need to be explored to know if these results can be generalized to short-term memory tasks using different paradigms or stimuli. Other studies used recognition memory tasks to show that pupil responses are different on trials with presentation of previously encoded items relative to new ones^12–15^, confirming their proposed role in memory processing. We observed pupil responses in the absence of visual stimulation during recall and no consistent responses to the countdown numbers presented on the screen. Therefore, these pupil responses were not driven by visual stimulation, suggesting that other sensory modalities of the presented stimuli, e.g. auditory tones, could induce similar responses. Modality-independence would be particularly important for applying pupil responses in memory enhancement technologies to trigger modulation of brain activity. For instance, pupil size can provide a non-invasive biomarker for brain stimulation during predicted states of poor memory encoding. Using pupil dilation to trigger brain stimulation would also provide a direct test of the relationship with memory processing and the underlying brain activity. In this study we have not explored other potential eye-tracking biomarkers of memory processing like the duration of fixations or the rate of saccades to the visual targets. In general, these and the electrophysiological measures remain to be compared in a range of task paradigms to draw further conclusions about the biomarkers of memory processing. Knowledge from combined recordings of brain activity and eye responses can be directly implemented into the emerging neuromodulation technologies.

## Methods

### Memory task

Ten healthy human subjects (five males) of age 20–37 years were recruited to a free recall verbal memory task with eye tracking. First six subjects were tested at the Mayo Clinic in Rochester MN, USA, and the last four subjects were tested at the Czech Technical University in Prague, Czech Republic. All subjects were fluent English speakers. Informed consent was obtained from all subjects and the study was conducted according to the institutional guidelines. Experimental protocol for testing memory was approved by the institutional review board at Mayo Clinic and the i4 tracking system for approved for use in human subjects. The task was based on classic paradigms for probing verbal memory^19^, in which subjects learned lists of words for a subsequent recall. Subjects were instructed to study lists of individual words presented sequentially on a laptop computer screen for a later memory test. Lists were composed of twelve words chosen at random from a pool of three hundred high frequency nouns (http://memory.psych.upenn.edu/WordPools). Each word remained on the screen for 1600 ms, followed by a 1000 ms blank interval between stimuli. Immediately following the final word in each list, participants performed a distractor task consisting of a series of arithmetic problems of the form ‘A + B + C = ??’, where A, B and C were randomly chosen integers ranging from 1–9. Following the distractor task participants were given 30 seconds to verbally recall as many words as possible from the list in any order. Vocal responses were digitally recorded by the laptop computer and later manually scored for analysis. Each session consisted of seventeen lists of this encoding-distractor-recall procedure.

### Tracking of eye movements and pupil dilation

Recording of gaze position and pupil size was performed using the ‘i4tracking’ system (Medicton group Inc.) designed for clinical applications in patients^20^. The recording was performed on a laptop computer connected to a 24-inch monitor screen with resolution of 1680 × 1050, where the gaze position was tracked by high-resolution (2048 × 1088) and high-speed (up to 200 Hz) external camera device. Stimuli were displayed on the screen using font size of 100 and were viewed from a distance of approx. 60 cm. Pupil position and size were detected by the camera device, corresponding to approx. 0.1 mm per pixel in the eye image. The camera device was placed below the screen to capture the face area from forehead to the mouth. Two sources of infrared light were emitted from the camera to capture the reflected light for pupil detection. Raw images from the camera were sampled at the rate of 50 Hz and were saved for extracting pupil using detection algorithms. The algorithms worked by fitting a general ellipse equation over the estimated pupil image. The pupil size in pixels was also converted to millimeters using estimated interpupillary distance (IPD) and the IPD in the camera images. The reported pupil area was computed as an average from both left and right eye using the corresponding vertical and horizontal diameters in ellipse area equation. Gaze position was determined by projecting the movement of the estimated center of the pupil onto the monitor screen area with the use of corneal reflection. Gazes outside of the screen area as well as the eye-blinks were treated as missing-samples. For further analysis, they were filled-in through linear interpolation between the closest samples at each end of the gap to obtain uninterrupted pupil size signal. The total blinking time was determined for each subject and was found to be less than 5% of the total recording time. Vocal responses of the subjects during the recall phase of the task were recorded using a built-in laptop microphone and manually annotated after the experiments in custom software for audio editing.

Before presentation of the task word lists, the eye tracker was calibrated for each recruited subject. In the calibration procedure subjects were asked to focus their gaze on nine points presented consecutively at specific positions across the diagonals and centers of the side edges of the display screen. Calibration was repeated throughout the session to ensure accurate estimate of the pupil size. Moreover, subjects were instructed not to move their heads and focus gaze on the screen throughout all phases of the task trials (Fig. 1). This was controlled and quantified by calculating the proportion of time spent gazing outside of a virtual rectangle surrounding the presented word (1.5 times the size of the word −700 × 200 pixels). All subjects spent negligible amount of time (<5%) blinking or gazing outside of center rectangle during the encoding phase. Only subject 4 spent more than 30% of the time gazing outside of the rectangle area during the recall phase and had to be excluded from the recall phase analysis (Fig. 2). All stimuli were presented on the screen in a light gray color on white background to minimize pupil responses to changing lighting and contrast. The testing was conducted in a room with low-light conditions that remained constant during the testing procedure.

### Analysis of pupil responses

Eye blinks were determined by comparing the output of the eye-tracker detection algorithm and three samples preceding and following any missing-value (~60 ms), which were used to interpolate the estimated pupil size and position during blinking, as described above. Proportion of the gaze focus outside of the screen center, where the stimuli were presented, was computed by dividing the total time outside of the rectangular area centered in the middle of the screen by the total time of uninterrupted eye-tracking without blinking. Pupil size was quantified as the raw recording of the pixel area (Figs 1–3) and also as estimated real area in square millimeters in individual subjects (Fig. 2). For comparisons across different subjects, the raw pupil area was normalized using a z-score transformation by expressing every sample as a standard deviation score from the mean calculated within each word list trial. Average estimates of the normalized pupil size were determined in 12-second time bins of the different phases of the task (Fig. 1) for statistical comparison. Likewise average estimates of the pupil area were determined in the ‘during recall’ epochs surrounding the onset of word vocalization (±1 second before and after the estimated 1-second vocalization time) to compare them to the remaining ‘outside recall’ epochs, which were outside of the vocalization epochs (Fig. 2). Average values of the mean, peak and trough in the pupil response of every subject were determined in two intervals of the encoding phase: ‘before’ and ‘after’ the word presentation from −200 ms to 0 ms from the onset and from 1000 ms to 1400 ms after the onset, respectively, for comparison between the recalled and forgotten word conditions (Fig. 3).

### Statistical analysis

All pupil size data were normalized using the z-score transformation given the approx. normal distribution of the data values in every subject. Two-way ANOVA was used to test the effect of different task phases and subjects on pupil size, which was followed by Tukey-Kramer post-hoc comparison of specific groups (Fig. 1). Paired t-test was used for all the remaining group comparisons of samples taken from the same trial (Figs 2b and 3a) or subject (Figs 2c and 3d). Bonferroni correction of the p-value was applied for the comparisons of mean and peak/trough values in the two time bins before and after the onset of word presentation (Fig. 3d). All results are presented as mean ± S.E.M.
