# Author Correction: Pupil size reflects successful encoding and recall of memory in humans

**DOI:** 10.1038/s41598-019-53308-w

**Published:** 2019-11-06

**Authors:** Michal T. Kucewicz, Jaromir Dolezal, Vaclav Kremen, Brent M. Berry, Laura R. Miller, Abigail L. Magee, Vratislav Fabian, Gregory A. Worrell

**Affiliations:** 1 Department of Neurology, Mayo Clinic, Rochester, MN USA; 2 Department of Physiology & Biomedical Engineering, Mayo Clinic, Rochester, MN USA; 3 Czech Institute of Informatics, Robotics and Cybernetics, Czech Technical University in Prague, Prague, Czech Republic; 4 Department of Physics, Faculty of Electrical Engineering, Czech Technical University in Prague, Prague, Czech Republic

Correction to: *Scientific Reports* 10.1038/s41598-018-23197-6, published online 21 March 2018

In the original version of this Article, Vratislav Fabian was incorrectly affiliated with ‘Czech Institute of Informatics, Robotics and Cybernetics, Czech Technical University in Prague, Prague, Czech Republic’. The correct affiliation is listed below.

Department of Physics, Faculty of Electrical Engineering, Czech Technical University in Prague, Prague, Czech Republic

This error has now been corrected in the PDF and HTML versions of the Article.

